# Expressive writing as a therapeutic intervention for people with advanced disease: a systematic review

**DOI:** 10.1186/s12904-019-0449-y

**Published:** 2019-08-02

**Authors:** N. Kupeli, G. Chatzitheodorou, N. A. Troop, D. McInnerney, P. Stone, B. Candy

**Affiliations:** 1 Marie Curie Palliative Care Research Department, Division of Psychiatry, University College London, 6th Floor, Maple House, 149 Tottenham Court Road, London, W1T 7NF UK; 2 Department of Psychology, City University of London, London, UK; 3 Department of Psychology and Sport Sciences, University of Hertfordshire, Hertfordshire, UK

**Keywords:** Expressive writing, Emotional disclosure, Advanced disease, Palliative care, Psychological changes, Linguistic analyses, Systematic review, Meta-analyses

## Abstract

**Background:**

Expressive writing involves writing about stressful or traumatic experiences. Despite trials in people with advanced disease, no systematic review to date has critiqued the evidence on expressive writing in this population. To synthesise the evidence of the effects of expressive writing on pain, sleep, depression and anxiety in people with advanced disease.

**Methods:**

A systematic review according to the Preferred Reporting Items for Systematic Reviews and Meta-Analyses guidelines. CINAHL, CENTRAL, PsycINFO and PubMed were searched from January 1986 to March 2018. Other sources included clinical data registers and conference proceedings. Studies were included if they were randomised controlled trials that assessed the impact of an intervention involving expressive writing for adults with advanced disease and/or studies involving linguistic analysis on the expressive writing output. Methodological quality was assessed using the Cochrane risk of bias tool and the Mixed Methods Appraisal Tool. The Grading of Recommendations Assessment, Development and Evaluation tool was used to assess the level of evidence for the outcomes of interest. The protocol of this systematic review has been registered on PROSPERO (CRD42017058193).

**Results:**

Six eligible studies with a total of 288 participants were identified, including four randomised controlled trials. All of the trials were in cancer and recruited predominantly women. None of the interventions were tailored to the population. Studies had methodological shortcomings and evidence was generally of low quality. Combined analysis of the four trials, involving 214 participants in total, showed no clear difference in the effect of expressive writing on sleep, anxiety or depression compared to an active control. Pain was not evaluated in the trials. In contrast, analysis of the four studies that included linguistic analysis alluded to linguistic mechanisms for potential effects.

**Conclusion:**

Although the trial results suggest there is no benefit in expressive writing for people with advanced disease, the current evidence is limited. There is a need for more rigorous trials. It would be of benefit first to undertake exploratory research in trial design including how best to measure impact and in tailoring of the intervention to address the specific needs of people with advanced disease.

**Trial registration:**

The protocol of this systematic review has been registered on PROSPERO, which can be accessed here (registration number: CRD42017058193).

**Electronic supplementary material:**

The online version of this article (10.1186/s12904-019-0449-y) contains supplementary material, which is available to authorized users.

## Introduction

People living with progressive advanced chronic diseases, such as those at an advanced stage of cancer or renal failure, experience a decline in physical and psychosocial functioning and an increasing need for care and support. They often report sleep problems, low mood and anxiety [1, 2]. Moreover, people with advanced disease are a clinical population with complex needs and commonly report intense fear and thoughts about their eventual death and loss of hope [3]. There are various, often complex, multicomponent, therapeutic approaches available such as psychosocial interventions [4, 5], supportive-expressive therapy [6] and legacy leaving interventions [7]. Evidence suggests that these types of interventions may enhance well-being and other outcomes in people with advanced disease [7–9]. These interventions, however, have been under-evaluated in this population, and some are potentially costly to implement, as they require specialist personnel providing support over several months. This is perhaps reflected in varied, scant provision and limited practice guidelines [10]. Palliative care clinicians also report that they have limited access to referring patients to psychological services [11]. Moreover, with increasing pressure on service funding, there is an even greater need for evidence of intervention benefit [12].

Expressive Writing (EW), is a simple, potentially inexpensive, therapeutic intervention which involves writing daily for 15–20 min over 3–4 consecutive days [13]. EW can be completed at home [14] without the need for facilitation, a specialist therapist or a dedicated facility. It is an approach that has been found in primary research to aid the healing process following a traumatic experience and can bring about a reduction in biological indicators of stress and stress-related illness [15, 16]. EW has also been shown in individual studies to produce positive health benefits for clinical populations including effects on blood pressure [17], immune function [18], wound healing [19], sleep [20], depression [21], and pain [22]. It has been shown to serve as a significant means for people to remain socially active and to seek social support [23]. In people with advanced disease, the effects of EW are yet to be explored at review level. However, there are recent systematic reviews of trials in progressive and long-term conditions, many of which are in cancer patients (irrespective of disease stage) and survivors. Their findings on the impact of EW are mixed [24–27]. None of these reviews conclude that this intervention does not work in these populations, providing a reason to look further at trials in advanced disease. Instead they highlight that the intervention has not been sufficiently evaluated in high quality large trials. The reviews also point to challenges they found reviewing impact across trials because of heterogeneity in intervention and comparison arm protocol, outcome measurements and population groups. They raise concerns about the limited consideration given by trialists to tailoring the intervention to the specific needs of the population, or subgroups of the population in question (e.g., stage of disease). This is particularly relevant given the significant differences in physical capability (e.g., ability to comfortably complete a writing session) and psychological pressures (e.g., fears about approaching death) that are likely to exist between those living with early stage or chronic illness compared to those with advanced or terminal disease. They also question the consideration of the size of impact which whilst small may be clinically relevant in such ill populations. All recommend further studies of rigorous design to determine whether it is therapeutically effective. One also highlights the potential underuse of linguistic analysis in evaluation of impact [24].

There are a number of theories about the processes that underlie EW [28]. For example, the psychosomatic theory of inhibition combines the notion of emotion inhibition with an element of cognitive processing [15]. This theory suggests that the act of inhibiting thoughts and feelings surrounding a traumatic experience requires physiological effort [15]. The physiological energy used to store and conceal personal trauma can result in rumination about the event and the accumulation of stress can lead to a higher rate of disease. However, this account of expressive disclosure relies on recounting and reappraising relatively inhibited trauma. Research suggests that describing emotions surrounding an imaginary event or a positive experience can also have health benefits [29–31]. A more plausible explanation is the emotion regulation view of EW, which takes into account the role of mastery in managing emotions, physiological responses and behaviours associated with the trauma [31, 32]. This interpretation suggests that the therapeutic element of emotional arousal following expressive disclosure is the important component, rather than the stimulus that produced the response. Thereby, disclosure functions as an affect regulation process, with the act of confronting thoughts and feelings associated with trauma improving perceptions of control and self-efficacy over the negative emotions experienced as a result of adversity.

Using the Linguistic Inquiry and Word Count (LIWC [33]) tool, the writing of expressive writers can also be analysed to provide insight into how language use might be linked with health benefits and the potential mechanisms driving EW [34–36]. The LIWC is a software program designed to evaluate whether and how language use might be linked with improved health. LIWC groups words into various categories such as grammar and parts of speech (e.g., pronouns, verbs, tense) and psychological (e.g., positive and negative emotions, achievement), social and cognitive processes (e.g., causation and insight) [37]. It is suggested that through the act of writing, cognitive changes occur as individuals organise their thoughts and feelings into a coherent narrative and understand better their negative experiences [38]. This is evident by the increased use of causal and insight words, indicating that expressive writers are attempting to find meaning in their experience [38].

Despite a number of studies, a preliminary scope of the literature found that to date no systematic review has evaluated the current evidence on efficacy of EW in adults with advanced disease. Thus, this review will examine whether EW could be beneficial for this clinical population with specific physical and psychological needs. Taking the results of reviews in broader populations, we may find clearer results in this more focused population. Moreover, to enhance critique of the evidence, the review will draw on the authors conclusions of these broader reviews in exploring, for instance, linguistics and whether there was tailoring of the intervention to the population. The aims of this systematic review are to:

1. Critique the trial evidence on EW as a therapeutic intervention for adults with advanced disease.
2. Explore the linguistic analysis of the participants’ EW in order to identify potential processes by which benefits occur.

## Method

The protocol for this review is registered on PROSPERO (CRD42017058193) and follows the Preferred Reporting Items for Systematic Reviews and Meta-Analyses (PRISMA [39]) (See Additional file 1: Table S1).

### Inclusion criteria

The type of studies selected was based on the two objectives of the systematic review [1]; RCTs and other comparative studies such as non-randomised experiments and before-and-after studies were eligible and [2] studies which reported the results of linguistic analysis.

Study participants were adults (aged 18 or over) with a diagnosis of advanced disease such as advanced/metastatic cancer and/or treated with a palliative care intent. Studies with children or patients without a diagnosis of advanced disease and/or patients being treated with a curative care intent were not included. As it was envisaged that there may be few studies whose samples completely fulfilled this criteria it was decided to also include studies whose samples consisted of > 50% advanced stage disease patients. Where such studies were identified, we sought to report findings only on the subgroup with advanced disease. If this was not possible, we reported findings for the whole group but take into account the limitations of doing this in our conclusions.

Studies which used EW as a structured therapeutic intervention were included. Studies were eligible if they used emotion provoking EW tasks as a method of relieving psychological or physical symptoms experienced by people with advanced disease. Studies with EW tasks which were not designed to be emotionally arousing or studies which included EW as part of a group therapy or as a psychotherapeutic intervention were not eligible. Trial suitable comparators were writing tasks that were non-emotionally arousing, no EW task, or treatment as usual.

Case studies, studies with a diary format and qualitative studies were excluded. Studies that assessed the value of EW for family carers were not included.

### Outcomes of interest

Our primary outcomes of interest were pain, sleep, depression and anxiety. They were selected because they are relevant to a population with advanced disease and they are commonly measured outcomes for therapeutic interventions in palliative care (e.g. [8, 9]). Also individual studies in other populations has found that EW has a positive impact on these outcomes [20–22, 40].

### Search strategy

Electronic bibliographic databases including the Cumulative Index to Nursing and Allied Health Literature (CINAHL), Cochrane Central Register of Controlled Trials (CENTRAL), PsycINFO and PubMed were searched from 1986 to March 2018. A variation of Medical Subject Headings (MeSH) terms and free-text terms on emotional disclosure and end-of life were applied. The emotional disclosure terms were adapted from a similar review [24] and end-of-life terms recommended by the Cochrane Palliative Care research group were used. Studies were included if they were published in English and after 1986 to reflect studies conducted after the original paradigm was developed [13]. Table 1 presents an example of the search strategy string used for the PsycINFO database, which was then adapted and applied to the remaining databases.

**Table 1:** Search strategy string for PsycINFO database

Story writing OR written paradigm OR descriptive writing OR emotional disclosure OR written emotional disclosure OR emotional expression OR illness narrative OR self-disclosure OR Pennebaker OR express* OR expressive writing OR writ* OR writing cure OR creat* OR reflect* OR catharsis OR trauma OR diary OR therapeutic writing OR therapeutic disclosure OR diary keeping	
AND	
((palliat* or terminal* or endstage or hospice* or metastatic or (end adj3 life) or (care adj3 dying) or ((advanced or late or last or end or final) adj3 (stage* or phase*))))	

*Note.* * = truncation symbol in order to find variations and plurals of words

The European Union clinical trials register, clinicaltrials.gov, the European Association for Palliative Care (EAPC) conference abstract proceedings for the last six years (2012–2017) and reference lists of included studies and relevant review articles were also checked to identify additional citations. A research interest list compiled by the British Psychological Society (BPS) was also used to contact other researchers who had expressed an interest in this field to find out if they were aware of any studies which may be relevant to this review.

### Study selection

The process of selecting relevant studies was conducted by two independent reviewers for all citations (GC and NK). Firstly, titles and abstracts were assessed using the inclusion criteria. The full-text review process was completed for studies that met the inclusion criteria or for studies with unclear relevance. Study authors were contacted if the relevance of a paper was unclear. Any disagreements in eligibility criteria that arose between the two reviewers were resolved through discussion and consensus. A third reviewer (BC) was consulted if discrepancies were unresolved.

### Data extraction

Data were extracted from each study using a standardised form developed by two reviewers (GC and NK). One reviewer (GC) completed the data extraction process in full. A second reviewer (NK) checked the extracted data and disagreements were resolved with the contribution of a third reviewer (BC). Data extraction included study characteristics (author, year, country), aim of the study, patient characteristics (gender, ethnicity, patient population, care setting), sample size, tasks (characteristics of the experimental and control tasks), and follow-up assessments. Outcome data such as risk ratios, means and standard deviations were extracted or generated if appropriate for all outcomes of interest. Additional information was requested from authors where necessary.

### Quality assessment

Two methodological quality assessment tools were used to examine the included studies. Comparative studies were assessed using the Cochrane Collaboration’s risk of bias tool [41]. The criteria assessed eight domains: selection bias, (random sequence generation and allocation concealment), performance bias (blinding of participants and personnel), detection bias (blinding of outcome assessment), attrition bias (incomplete outcome data) and sample size. Each domain was rated as either high risk, low risk or unclear risk of bias.

The Mixed Methods Appraisal Tool (MMAT [42]) was used to assess the quality of the studies which did not employ an RCT design. Each study was assessed on study design, selection bias, measurement appropriateness, participant comparability, sampling strategy, representativeness of the sample, complete outcome data and response rate. An overall quality percentage and a descriptive score was calculated for each of the studies according to the number of criteria met. The total score was calculated using the total number of criteria that the studies met divided by four. Scores ranged from 25 to 100%. Two independent reviewers completed the Cochrane risk of bias assessment and the MMAT tool. Reviewers met to compare the quality appraisal ratings and consensus was reached through discussion.

The evidence for each primary outcome of the included RCTs was assessed by the Grading of Recommendations Assessment, Development and Evaluation (GRADE) system [43]. GRADE does not judge the quality of the individual studies, but takes into account the results across the studies and from any combined analyses [44]. The GRADE approach incorporates criteria for downgrading the quality of evidence derived from trials [45]. When the grade of evidence is judged as low, this indicates that future research *might* change the estimate of effect. A very low grade of evidence indicates that it is *likely* the estimate of effect is markedly different from the true effect. The quality of evidence is downgraded in the presence of study limitations (risk of bias), imprecision (small sample size, width of confidence intervals), indirectness (comprehensive population, intervention, control, and outcomes criteria; population, intervention, control, and outcomes found across trials to have extreme differences in direction of results [PICO]), publication bias and inconsistency (over 80% statistical heterogeneity in outcomes, interventions).

### Data analysis/synthesis

For reported dichotomous data the risk ratios (RRs) and their confidence intervals (CI) were extracted or generated if appropriate data were provided in the study. For reported continuous data the mean difference (MD) and the standard deviation (SD) were extracted or computed if appropriate from the extracted data. If three or more studies were identified with data for one of our outcomes of interest, a meta-analysis was considered based on sufficient homogeneity in key characteristics across studies using the Review Manager 5.3 tool [46]. For any combined analyses, statistical heterogeneity was calculated with the I^2^ measure provided by the Review Manager 5.3 tool [46]. Allowing for normal distribution of the studies’ outcomes, a random-effects model was then used to combine data across studies found to have substantial statistical heterogeneity (> 50%) [47]. Conversely, allowing for power detection of the studies’ outcomes, a fixed-effects model was used for studies which had a < 50% statistical heterogeneity. For the second objective of the review, findings from the included studies were explored narratively using the data on word use and whether use of particular categories of words (i.e., emotion, cognitive) was associated with improvements in the outcomes of interest.

## Results

### Search results and study selection

**Fig. 1:**
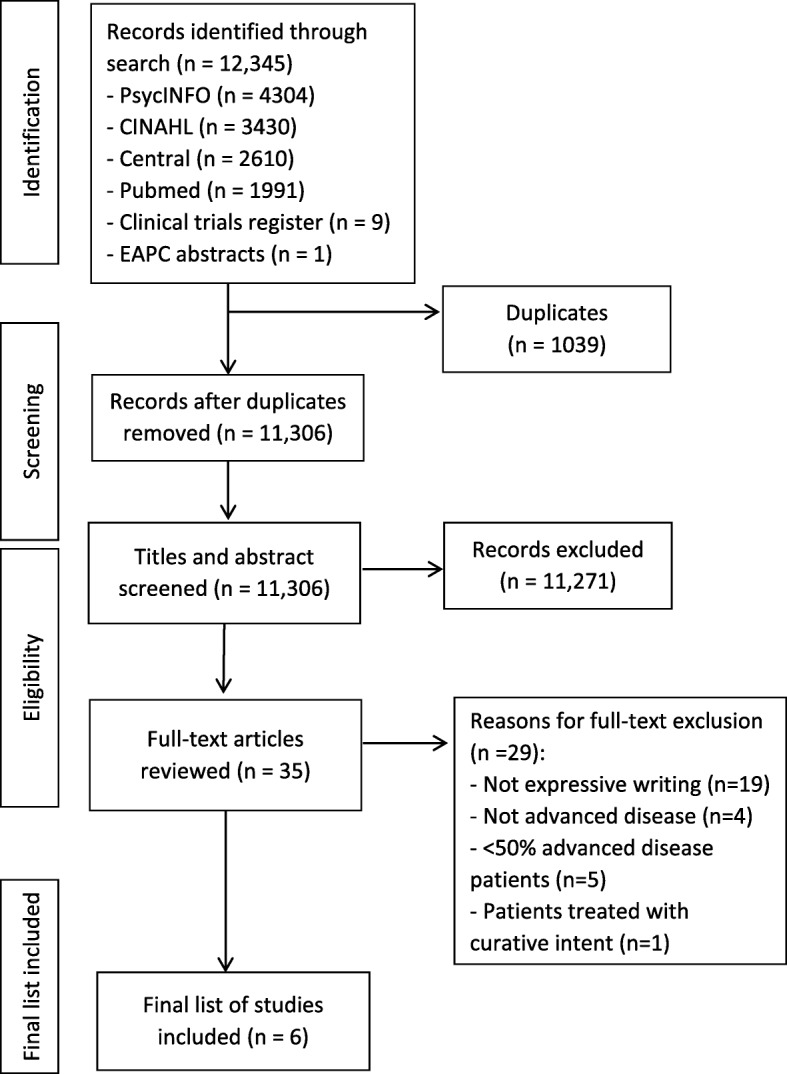
PRISMA flow diagram of study

Figure 1 presents the study selection process. The search yielded 11,306 unique citations. Following screening, 35 full-text papers were reviewed for potential eligibility and 29 were excluded. The reasons for exclusion were interventions that did not use EW (*n* = 19), studies with < 50% of participants with advanced disease (*n* = 5) or a sample of patients not diagnosed with advanced disease (*n* = 4), or patients who were being treated with a curative intent (n = 1). Six studies met the inclusion criteria. Four of them were RCTs [20, 48–50]. One of the other studies employed a non-randomised experiment [51] and the other reported the results of linguistic analyses of expressive writers’ entries from an RCT [52]. These were included in the linguistic analyses along with de Moor, Sterner [20] and Mosher, DuHamel [50].

### Study characteristics

Two tables summarise the characteristics of the included studies. Table 2 provides summaries of the four RCTs [20, 48–50]. Table 3 provides summaries of studies with linguistic analysis [20, 50–52].

**Table 2:** Summary of RCTs examining the effectiveness of an EW task in palliative care/advanced disease samples

Study characteristics	Setting, task and assessments	Measures and results
Bruera (2008)* *N* = 24 Population: Advanced Cancer Gender: *EW*: 75% female; *Control*: 66% female Median age: *EW*: 55.4; *Control*: 54.3 Ethnicity: *EW*: 92% White, 8% African American; *Control*: 58% White, 25% African American, 17% Hispanic	Setting: Inpatient and outpatient palliative care clinic Task: *EW*: Trauma (*n* = 12); *Control*: everyday behaviours and habits (n = 12) Four sessions for 20 min over two weeks Assessments: Baseline and before and after each writing session	Measures: STAI assessed anxiety levels before and after each writing session Results: STAI median scores (range): *EW:* Pre-writing State Anxiety = 34.5 (25–41); Post-intervention State Anxiety = 40 (26–44); *Control:* Pre-writing State Anxiety = 35 (20–48); Post-intervention State Anxiety = 37 (20–44)
De Moor (2002) *N* = 42 Population: Renal cell carcinoma Gender: 85.7% male Mean age: 56.4 Ethnicity: Not reported	Setting: Lab-based Task: *EW:* Cancer-related emotions (*n* = 21); *Control:* Health behaviours (n = 21) Four sessions for 20 min over four weeks Assessments: Immediately post-intervention and at four, six, eight, and 10 weeks	Measures: *PSQI* assessed sleep quality; *POMS* assessed depression, anxiety and fatigue; *IES* assessed distress Results: Follow-up scores showed that the EW group (M = 6.8, SE = 0.6) reported less sleep disturbance at follow-up compared with the control group (M = 8.7, SE = 0.7) but no differences were found for depression (EW: M = 7.4, SE = 1.1; Control: M = 6.6, SE = 1.2), anxiety (EW: M = 6.9, SE = 0.8; Control: M = 7.0, SE = 0.9), distress (EW: M = 17.4, SE = 1.7; Control: M = 14.6, SE = 1.8) and fatigue (EW: M = 9.5, SE = 1.0; Control: M = 9.6, SE = 1.1)
Low (2010) *N* = 62 Population: Metastatic breast cancer Gender: All female Mean age: 53.8 (SD = 10.8, range = 29–78) Ethnicity: 87% White	Setting: Home based Task: *EW:* Cancer-related emotions (*n* = 31); *Control:* Facts about cancer diagnosis and treatment (n = 31) Four sessions for 20 min over three weeks Assessments: Baseline and three months post-intervention	Measures: *PSQI* assessed sleep quality; *CES–D* assessed depression; *IES* assessed distress Results: Follow-up scores showed no differences between the EW and control groups for sleep (EW: M = 7.1, SE = 0.51; Control: M = 6.6, SE = 0.51), depression (EW: M = 12.8, SE = 1.47; Control: M = 13.2, SE = 1.48) and distress (EW: M = 8.7, SE = 0.94; Control: M = 10.1, SE = 0.96)
Mosher (2012) *N* = 86 Population: Advanced breast cancer Gender: All female Mean age: *EW:* 57.4 (SD = 12.5); *Control:* 58.5 (SD = 11.7) Ethnicity: 81.4% White, 7% African American, 5.8% Hispanic, 5.8% Other	Setting: Home based Task: *EW:* Cancer-related emotions (*n* = 44); *Control:* Previous day’s activities (*n* = 42) Four sessions for 20 min over four-seven weeks Assessments: Eight weeks post-intervention	Measures: *PSQI* assessed sleep quality; *CES-D* assessed depression; *HADS-A* assessed anxiety; *DT* assessed general distress; *FACIT-F* assessed fatigue Results: Follow-up scores showed no differences between the EW and control groups for sleep (EW: M = 8.42, SE = 0.39; Control: M = 7.83, SE = 0.39), depression (EW: M = 17.99, SE = 1.35; Control: M = 17.87, SE = 1.38), anxiety (EW: M = 7.15, SE = 0.48; Control: M = 7.87, SE = 0.49), distress (EW: M = 4.53, SE = 0.36; Control: M = 4.37, SE = 0.37) and fatigue (EW: M = 30.38, SE = 1.17; Control: M = 32.58, SE = 1.20)

*Note.* * = study not included in the meta-analysis; CES-D = Center for Epidemiologic Studies-Depression Scale [53]; DT = Distress Thermometer [54]; EW = Expressive Writing; FACIT-F = Functional Assessment of Chronic Illness Therapy-Fatigue [55]; HADS-A = Hospital Anxiety and Depression Scale [56]; IES = Impact of Events Scale [57]; M = Mean; POMS = Profile of Mood States [58]; PSQI = Pittsburgh Sleep Quality Index [59]; SS = Standard Error; STAI = State-Trait Anxiety Inventory [60]

**Table 3:** Summary of studies reporting linguistic analyses

Study characteristics	Setting, task and assessments	Measures and results
De Moor (2002) Design: RCT N = 42 Population: Renal cell carcinoma Gender: 85.7% male Mean age: 56.4 Ethnicity: Not reported	Setting: Lab-based Task: *EW:* Cancer-related emotions (n = 21); *Control:* Health behaviours (n = 21) Four sessions for 20 min over four weeks Assessments: Immediately post-intervention and at four, six, eight, and 10 weeks	Linguistic analyses: conducted using the LIWC Results: EW and control groups differed in the words they used for 24 of the 32 categories suggesting emotional and cognitive processing and expression of their cancer experience
Imrie & Troop (2012) Design: Non-randomised experiment N = 6 Population: Secondary cancer or life-limiting illness Gender: 61.5% female Mean age: 67.5 (SD = 14.9; range = 38–86) Ethnicity: Not reported	Setting: Day Hospice Task: *EW:* Difficult experience from the previous week followed by expressing compassion for the self in the entry (n = 3); *Control:* Difficult experience from the previous week (n = 3) Three sessions for 20 min over three weeks Assessments: Baseline and one-week post-intervention	Linguistic analyses: conducted using the LIWC Results: Both groups reduced the number of negative words they used between baseline and follow-up (*F*_1,18_ = 6.97, *p* < 0.02) but compared to the control group, the expressive writers increased the number of causal words used over time (*F*_1,18_ = 8.36, *p* < 0.01)
Laccetti (2007) Design: Secondary analysis of EW entries from RCT *N* = 68 Population: Metastatic breast cancer Gender: All females Mean age: 51 (range = 36–78) Ethnicity: 94% White, 5% Native American, 1% Other	Setting: Outpatient clinics Task: *EW:* Four sessions for 20–30 min over four consecutive days about experiences, thoughts and feelings related to not fully recovering from cancer and facing death, and traumatic and upsetting experiences in life that may or may not relate to cancer Assessments: Within one week of study entry and three months post-intervention	Linguistic analyses: conducted using the LIWC Measures: *FACT-B* assessed quality of life Results: Expressive writers who used more positive emotion words reported higher scores on emotional well-being (β = 1.87 [95% CI 0.33, 3.42], *p* = 0.02) and concerns related to their breast cancer of FACT-B (β = 1.75 [95% CI 0.17, 3.33] *p* = 0.03) three months post-intervention compared to those who used more negative emotion words
Mosher (2012) Design: RCT N = 86 Population: Advanced breast cancer Gender: All female Mean age: *EW:* 57.4 (SD = 12.5); *Control:* 58.5 (SD = 11.7) Ethnicity: 81.4% White, 7% African American, 5.8% Hispanic, 5.8% Other	Setting: Home based Task: *EW:* Cancer-related emotions (n = 44); *Control:* Previous day’s activities (*n* = 42) Four sessions for 20 min over four-seven weeks Assessments: Eight weeks post-intervention	Linguistic analyses: conducted using the LIWC Results: EW group used a higher proportion of positive (η^2^*p* = 0.13, *p* < 0.001) and negative (η^2^*p* = 0.46, *p* < 0.001) words compared to the control group

*Note*. EW = Expressive Writing; FACT-B = Functional Assessment of Cancer Therapy – Breast [61]; LIWC = Linguistic Inquiry and Word Count

All studies were published between 2002 and 2013. All but one study (United Kingdom; Imrie and Troop [51]) was conducted in the USA. Sample sizes ranged from 6 to 86 participants. In total, across the studies, there were 288 participants. Participants’ diagnoses were metastatic/advanced breast cancer [49, 50, 52], advanced cancer [48] and renal-cell carcinoma [20]. One study had mixed sample participants with advanced stage disease including secondary cancer and life-limiting illness [51]. As per gender distributions, all but one study [20] included predominantly or exclusively female participants [48–52]. Mean age ranged from 51 to 58.5 years. Participants were predominantly white [48–50, 52], while ethnic composition was not reported in two studies [20, 51].

The settings where participants completed the writing tasks included at home [49, 50], at a day hospice [51], in a laboratory [20] and in hospital palliative care units [48, 52]. The number of intervention sessions, their duration and their administration intervals varied to some extent across the studies. All studies consisted of four writing sessions except for Imrie and Troop [51] which consisted of two sessions. The duration of the sessions, where this was reported, was between 20 and 30 min. The sessions were administered over four consecutive days [52], over two weeks [48, 51], over three weeks [49], over four weeks [20], and over four to seven weeks [50]. The outcome assessments for studies were performed at baseline and then immediately post-intervention [20, 48], one week post-intervention [51], eight weeks post-intervention [50], three months post-intervention [49, 52] and four, six, eight and ten weeks post-intervention (data averaged across five follow-up assessments [20]).

The majority of studies instructed participants to write about their deepest emotions, thoughts and feelings specifically surrounding their cancer experience [20, 49, 50, 52] or about a traumatic and upsetting or difficult event [48, 51]. Several different neutral prompts for the control task were used including writing about daily habits and activities [48, 50], health-related behaviours [20], and facts about their cancer diagnosis and treatment [49]. In contrast, one study used an emotionally-arousing task as a control condition by instructing participants to write about an event that took place the previous week which provoked worry [51] but this was to contrast with the self-compassion prompt used for the EW task.

### Risk of bias assessment of RCTs

Online Additional file 2: Table S2 summarises the quality appraisal of the included RCTs. Overall, the studies had an unclear risk of bias. Three of the four studies were found to be low risk for selection bias [20, 49, 50]. The allocation concealment was considered unclear in most studies except for Low, Stanton [49] which used sequentially numbered envelopes to conceal the allocation of tasks for each participant. The majority of the studies were graded as low risk for attrition except for Bruera, Willey [48] which reported a high drop-out rate (75%) and all studies were graded as high risk of bias for sample size due to small samples employed (< 50 per trial arm).

### Mixed methods appraisal tool (MMAT)

Online Additional file 3: Table S3 presents the quality appraisal of the two studies with multiple methods designs [51, 52]. The Imrie and Troop [51] study was rated at 75%, whilst the study by Laccetti [52] scored 50%. Research questions and objectives were clearly defined and stated in both studies while a common strength was that the measurements used were appropriate and validated. Imrie and Troop [51] did not appropriately discuss the random allocation process. On the other hand, sampling strategies were sufficiently detailed in the Laccetti [52] study. Regarding complete outcome data, Imrie and Troop [51] reported their response rates while Laccetti [52] failed to report them sufficiently.

### Effect of EW on primary and secondary outcomes

Included studies that reported outcomes for sleep, depression, and distress outcomes were suitable for pooling in combined analyses. A variety of self-report measures were used to assess outcomes including the Pittsburgh Sleep Quality Index (PSQI [59]) for sleep, Center for Epidemiologic Studies – Depression scale (CES-D [53]) for depression, the Distress Thermometer (DT [54]) for distress and the Profile of Mood States (POMS [58]) for anxiety and fatigue. The frequency and duration of EW was similar across the studies despite variations in the time period in which the tasks were completed. All studies reported continuous data. Online Additional file 4: presents the results of the pooled outcomes for sleep, depression and distress.

### Primary outcomes of interest: results and judgement of quality of results

#### Pain

Although two studies examined the effects of EW on pain, one study did not report the data on pain due to high attrition [48] and one study reported the effects of EW on a number of somatic symptoms including pain but did not report the effects on pain separately [49].

#### Sleep

Data from three studies measuring sleep were pooled with a total sample of 183 participants [20, 49, 50]. In the combined analysis from all time points (mean scores averaged across immediately post-intervention and four, six, eight and ten weeks post-intervention [20]; 3 months [49]; 8 weeks [50]) there was no statistically significant effect of EW on sleep between the trials arms with a standardised mean difference (SMD) of − 0.12 [95% CI − 1.51, 1.26]. The data showed high heterogeneity across the trials (I^2^ = 65%, *p* = 0.06). (See online Additional file 4: Figure S1).

#### Depression

As in the combined analysis at follow-up, three studies of 183 participants [20, 49, 50] demonstrated no statistically significant effect of EW on depression between the trials arms (SMD = 0.02 [95% CI -0.27, 0.31]). There was no evidence of heterogeneity among the trials (I^2^ = 0%, *p* = 0.88). (See online Additional file 4: Figure S2).

#### Anxiety

Two studies with a total of 121 participants both showed that the intervention had no statistically significant effect on anxiety (MD = -0.10 [− 2.46, 2.26] [20]; MD = -0.72 [95% CI -2.06, 0.62] [50]).

### Secondary outcomes of interest

#### Distress

As in combined analysis at follow-up, data from three studies with a total of 183 participants [20, 49, 50] showed no statistically significant improvements of EW on distress between the trial arms (SMD = -0.03 [95% CI -0.32, 0.26]). There was no evidence of statistical heterogeneity across the trials (I^2^ = 0%, *p* = 0.52). (See online Additional file 4: Figure S3).

#### Fatigue

Two studies with a total of 121 participants both showed that the intervention had no statistically significant effect on fatigue (MD = -0.10 [95% CI -3.01, 2.81] [20]; MD = -2.20 [95% CI -5.49, 1.09] [50]).

#### Grade

Overall, the quality of evidence for the primary outcomes was rated as low. For the primary outcomes of sleep, anxiety and depression the grade of evidence was downgraded due to some important factors. First, data from three studies [20, 49, 50] measuring sleep had inconsistencies as they were presented in combined analysis with substantial statistical heterogeneity (I^2^ = 65%). For the primary outcome of depression, data from the same three studies showed imprecision as they had small sample sizes and limitations to study quality due to unclear risk of bias. Finally, the evidence for the effectiveness of EW on anxiety from two studies was also rated as low as these studies showed imprecisions due to small sample sizes and unclear risk of bias due to underreporting [20, 50].

#### Linguistic analysis

Four studies used the LIWC [33] to analyse word use across EW text entries [20, 50–52]. While de Moor, Sterner [20] found that the use of cognitive words increased in the EW group, Imrie and Troop [51] found this was especially true in the use of causal words in those who were writing about stress with an additional self-compassionate component. There were also changes in the use of emotion words across text entries in the EW groups although the results here were more mixed. Mosher, DuHamel [50] found that the EW group used more positive and negative emotion words while Imrie and Troop [51] found a reduction in negative emotion words in both the control and EW groups. Laccetti [52] found that greater use of positive emotion words was related to improvement in quality of life. The EW task in the Mosher, DuHamel [50] study prompted the intervention group to express themselves using emotional words and thus the authors found that, compared with the control group, expressive writers used a higher percentage of both positive and negative emotion words.

## Discussion

This systematic review sought to critique evidence evaluating EW in advanced disease. All of the studies identified involved adults with advanced disease and from these, five, including the four RCTs, involved adults with advanced cancer. The results showed, on current evidence, that EW does not have a beneficial effect in people with advanced cancer in terms of primary outcomes of sleep, depression or anxiety or secondary outcomes of distress and fatigue. However, the evidence is based on only a few studies which have significant methodological limitations as judged using GRADE [43]. Notably, although two of the trials assessed the effects of EW on pain, they did not report the outcome of this assessment. Which is important considering that previous research with breast cancer survivors who completed an EW task reported a reduction in pain at the three-month post-intervention follow-up [21].

Notwithstanding the lack of evidence, these findings are consistent with studies which found no effect of EW on sleep [62] or anxiety [40]. The evidence for the effect of EW on depression is equivocal, as positive effects have been reported at three months but not at nine-month follow-up in breast cancer survivors [21]. On the other hand, perhaps in an advanced disease population it might be overly optimistic to expect an improvement in these outcomes, given the patients are extremely unwell and likely to have deteriorating health. We selected these outcomes based on knowledge elsewhere of their relevance. However this choice did not deviate from those used in the trials of populations with advanced disease. Few other outcomes such as quality of life or qualitative patient-reported opinion were measured in these trials. Perhaps future research should consider the types of outcomes that could be impacted by the intervention in this population.

The studies identified in this review used two to four sessions of EW over two to seven weeks which may not have been sufficient to produce beneficial effects. Thus a tailored EW intervention with an adequate but suitable and feasible number of sessions for people with advanced cancer is required. Moreover, the format of EW may not be appropriate for all patients in this population, where some may find the act of writing regularly too much to commit too and other easier formats such as recorded spoken expression may be more appropriate.

The synthesis of the linguistic analyses revealed that participants who completed the EW task used an increasing number of emotional words including both positive and negative emotion words [50] but it is the use of positive emotion words that is related to greater emotional well-being [52]. Moreover, expressive writers were found to use more cognitive words relating to causal understanding in their text entries suggesting cognitive changes [51].

### Strengths and weaknesses

The present review was conducted in accordance with the PRISMA criteria [39]. A comprehensive search for relevant studies was conducted using four electronic databases, clinical data registers, published conference abstract proceedings, reference lists of included studies, review articles and a research interest list compiled by the British Psychological Society (BPS) for additional citations. Authors of included studies were contacted for study clarifications and missing information and to enquire of any literature which may have been missed by the search process. In order to maximise the number of eligible studies, it was decided to include all studies which employed samples consisting of > 50% patients with advance disease. In fact, in all studies reviewed, all participants had advanced disease and results may therefore be representative of this population. The present review assessed the quality of studies and evidence using the Cochrane risk of bias assessment [41] and the GRADE [43] system, respectively. Finally, an important strength of the present review is the use of meta-analysis which provides a pooled estimate of EW effects. All available data were pooled after ensuring that the follow-ups and the outcome measures were similar enough to allow combined analyses.

This review has some potential limitations. Among the most important ones is that the sample sizes of the included studies were small, ranging from 6 to 86 participants. This indicates caution should be taken in interpreting the results as small studies may account for false-positive results [63]. Furthermore, some may argue that there was insufficient data to combine, however the combined analysis is not misleading as studies were sufficiently homogeneous and by using the GRADE system to judge quality of evidence, we highlight that we base our conclusions on limited evidence. The assessment follow-ups also varied between the studies from immediately post-intervention to three months with one study averaging the follow-up scores across assessments completed immediately post-intervention and at four, six, eight and ten weeks post-intervention [20]. Also, one study conducted basic statistical analyses comparing median scores by “eye-balling” the data [48]. After contacting the authors, they confirmed that this was due to insufficient power and high attrition. Therefore, further conclusions cannot be drawn from this study. Additionally, studies did not recruit people with other life-limiting conditions except for cancer and, as such, findings cannot be generalised to other advanced conditions. Moreover, there was a significant limitation with regards to the overall methodological quality of the included studies, which was considered to be unclear according to the Cochrane risk of bias tool, with the evidence also considered to be of low quality according to the GRADE approach.

### Implications for practice and research

EW is a low cost, relatively simple and easy intervention to administer. Where it has been shown to have significant beneficial effects, it is appealing to propose adding EW as a standalone intervention or an adjunct to existing services. The findings of this review suggest the evidence for the benefits of EW in people with advanced disease is simply not there at present. This though may relate to trial design and quality of execution. Moreover, since the pattern of word use is consistent with those studies that do show benefits [64] and that participants report EW as personal, meaningful and valuable [51], we argue that it is worth pursuing this line of research with better quality studies before reaching a final conclusion. Specifically, studies need to be sufficiently powered, to have enough writing sessions, and to have a long enough follow-up. Tailoring the methodology of emotional disclosure interventions to address the specific needs of people with advanced disease (e.g., in terms of frequency and location of sessions, precise disclosure instructions, type of practical and emotional support available and whether disclosure should be spoken or written) may improve its acceptability and potential usefulness with this population. Similarly, further exploration of outcome measurement focusing on what impact EW may have in this very ill population could help identify any potential benefit. A collaborative project funded by the Economic and Social Research Council and Marie Curie awarded to our team will be exploring these gaps in our understanding.

Moreover, even if a positive effect for EW (or other form of emotional disclosure intervention) can be identified, further research should investigate the way in which these interventions can be used to supplement existing therapies in palliative care settings with the aim to improve the overall well-being of people with advanced disease. Trial evidence to date, where the effect of psychological therapies have been reviewed in this setting, has been found to be unclear on impact [9] or limited in quality [8]. If emotional disclosure is to be included as a therapeutic intervention, either as a standalone intervention or as part of a multi-component treatment, then it is advisable that harmful effects of expressing emotions should also be assessed.

Notably, studies included in this review have recruited predominantly female participants. Whilst it was not possible to investigate gender as a potential moderator in this review, it would be useful for future research to explore whether males and females with advanced disease respond differently to emotional disclosure interventions. Reviews to date have found contradictory findings with Smyth [62] reporting that men experience more benefits than women whilst the findings of Reinhold, Bürkner [65] suggest that females benefit more from EW.

There may also be other outcomes that are important to measure and measures of outcomes that are sufficiently sensitive to detect small changes. As discussed above, pain was not adequately reported as a standalone outcome. However, there is some suggestion that EW was related to an increase in visits to mental health services [50] which has also been found in other EW studies such as for people following their first myocardial infarction [66]. Moreover, EW was more beneficial for those who reported low levels of social support at baseline [49] which is in line with findings that posit that EW benefits people with fewer opportunities for emotional support [67] and can even improve perceived levels of support in women with cancer [68]. These additional outcomes, if appropriately measured, may be important to consider. RCTs provide a quantitative measure of outcomes which may not assess what patients value or how they would like an EW intervention to be delivered. Therefore, quantitative data should be supplemented with qualitative data [69].

It is also worth focusing on the association between the use of positive words and improvements in well-being. It is recommended that more studies need to evaluate this association and to expand our understanding of the duration of the effects of emotional disclosure. Additionally, given the fact that the LIWC is unable to identify traces of irony or sarcasm, it perhaps needs to be considered how these paramount elements of language could be incorporated into the LIWC [33] or alternatively through coding of voice recordings by independent raters.

## Conclusion

The findings of this review highlight that the use of EW as a therapeutic intervention in people with advanced disease is feasible but that a more tailored, focused intervention may be required in order to improve outcomes. These then should be tested in high quality adequately powered studies.

## Additional files

Additional file 1: **Table S1**: PRISMA 2009 Checklist. (DOCX 27 kb)

Additional file 2: **Table S2**: Risk of bias assessment across RCTS. (DOCX 32 kb)

Additional file 3: **Table s3**: Results of MMAT quality appraisal. (DOCX 14 kb)

Additional file 4: Results of the meta-analysis for the outcomes of interest. **Figure S1.** Pooled data comparing the effectiveness of EW on sleep (sleep quality/sleep duration) compared with the control task. **Figure S2.** Pooled data comparing the effectiveness of EW on depression compared with the control task. **Figure S3.** Pooled data comparing the effectiveness of EW on distress compared with the control task. (DOCX 31 kb)

## Data Availability

The datasets used and/or analysed during the current review are available from the corresponding author on reasonable request.

## Abbreviations

EW: Expressive Writing
GRADE: Grading of Recommendations Assessment, Development and Evaluation
LIWC: Linguistic Inquiry and Word Count
MMAT: Mixed Methods Appraisal Tool
RCT: Randomised Control Trial

## References

[1] Mitchell AJ, Chan M, Bhatti H, Halton M, Grassi L, Johansen C (2011). Prevalence of depression, anxiety, and adjustment disorder in oncological, haematological, and palliative-care settings: a meta-analysis of 94 interview-based studies. The Lancet Oncology.

[2] Potter J, Hami F, Bryan T, Quigley C (2003). Symptoms in 400 patients referred to palliative care services: prevalence and patterns. Palliat Med.

[3] Ellis LM, Blanke CD, Roach N (2015). Losing “losing the Battle with Cancer”. JAMA Oncol.

[4] Badr H (2016). Psychosocial interventions for patients with advanced cancer and their families. Am J Lifestyle Med.

[5] von Heymann-Horan AB, Puggaard LB, Nissen KG, Benthien KS, Bidstrup P, Coyne J (2017). Dyadic psychological intervention for patients with cancer and caregivers in home-based specialized palliative care: the Domus model. Palliat Support Care.

[6] Spiegel D, Spira J (1991). Supportive-expressive group therapy: a treatment manual of psychosocial intervention for women with recurrent breast cancer: psychosocial treatment laboratory.

[7] Keall RM, Clayton JM, Butow PN (2015). Therapeutic life review in palliative care: a systematic review of quantitative evaluations. J Pain Symptom Manag.

[8] Okuyama T, Akechi T, Mackenzie L, Furukawa TA (2017). Psychotherapy for depression among advanced, incurable cancer patients: a systematic review and meta-analysis. Cancer Treat Rev.

[9] Beatty L, Kemp E, Butow P, Girgis A, Schofield P, Turner J (2018). A systematic review of psychotherapeutic interventions for women with metastatic breast cancer: context matters. Psycho-oncology..

[10] National Institute for Health and Clinical Excellence. End of life care for adults: Holistic support - families and carers 2017. Available from: https://www.nice.org.uk/guidance/qs13/chapter/Quality-statement-7-Holistic-support-families-and-carers. Accessed 28 Mar 2018.

[11] Atkin N, Vickerstaff V, Candy B (2017). ‘Worried to death’: the assessment and management of anxiety in patients with advanced life-limiting disease, a national survey of palliative medicine physicians. BMC Palliative Care.

[12] Gardiner Clare, Ryan Tony, Gott Merryn (2018). What is the cost of palliative care in the UK? A systematic review. BMJ Supportive & Palliative Care.

[13] Pennebaker JW, Beall SK (1986). Confronting a traumatic event: toward an understanding of inhibition and disease. J Abnorm Psychol.

[14] van Middendorp H, Sorbi MJ, van Doornen LJP, Bijlsma JWJ, Geenen R (2007). Feasibility and induced cognitive-emotional change of an emotional disclosure intervention adapted for home application. Patient Educ Couns.

[15] Pennebaker JW (1985). Traumatic experience and psychosomatic disease: exploring the roles of behavioural inhibition, obsession, and confiding. Canadian Psychology/Psychologie canadienne.

[16] Pennebaker JW (1997). Writing about emotional experiences as a therapeutic process. Psychol Sci.

[17] McGuire KMB, Greenberg MA, Gevirtz R (2005). Autonomic effects of expressive writing in individuals with elevated blood pressure. J Health Psychol.

[18] Petrie KJ, Fontanilla I, Thomas MG, Booth RJ, Pennebaker JW (2004). Effect of written emotional expression on immune function in patients with human immunodeficiency virus infection: a randomized trial. Psychosom Med.

[19] Weinman J, Ebrecht M, Scott S, Walburn J, Dyson M (2008). Enhanced wound healing after emotional disclosure intervention. Br J Health Psychol.

[20] de Moor C, Sterner J, Hall M, Warneke C, Gilani Z, Amato R (2002). A pilot study of the effects of expressive writing on psychological and behavioral adjustment in patients enrolled in a phase II trial of vaccine therapy for metastatic renal cell carcinoma. Health Psychol.

[21] Henry EA, Schlegel RJ, Talley AE, Molix LA, Bettencourt BA (2010). The feasibility and effectiveness of expressive writing for rural and urban breast Cancer survivors. Oncol Nurs Forum.

[22] Rosenberg HJ, Rosenberg SD, Ernstoff MS, Wolford GL, Amdur RJ, Elshamy MR (2002). Expressive disclosure and health outcomes in a prostate cancer population. Int J Psychiatry Med.

[23] Pennebaker JW, Graybeal A (2001). Patterns of natural language use: disclosure, personality, and social integration. Curr Dir Psychol Sci.

[24] Merz EL, Fox RS, Malcarne VL (2014). Expressive writing interventions in cancer patients: a systematic review. Health Psychol Rev.

[25] Zachariae R, O'Toole MS (2015). The effect of expressive writing intervention on psychological and physical health outcomes in cancer patients-a systematic review and meta-analysis. Psycho-Oncology..

[26] Nyssen Olga P, Taylor Stephanie JC, Wong Geoff, Steed Elizabeth, Bourke Liam, Lord Joanne, Ross Carol A, Hayman Sheila, Field Victoria, Higgins Ailish, Greenhalgh Trisha, Meads Catherine (2016). Does therapeutic writing help people with long-term conditions? Systematic review, realist synthesis and economic considerations. Health Technology Assessment.

[27] Zhou C, Wu Y, An S, Li X (2015). Effect of expressive writing intervention on health outcomes in breast cancer patients: a systematic review and meta-analysis of randomized controlled trials. PLoS One.

[28] Sloan DM, Marx BP (2004). Taking pen to hand: evaluating theories underlying the written disclosure paradigm. Clin Psychol Sci Pract.

[29] Burton CM, King LA (2009). The health benefits of writing about positive experiences: the role of broadened cognition. Psychol Health.

[30] Low CA, Stanton AL, Danoff-Burg S (2006). Expressive disclosure and benefit finding among breast cancer patients: mechanisms for positive health effects. Health Psychol.

[31] Greenberg MA, Wortman CB, Stone AA (1996). Emotional expression and physical health: revising traumatic memories or fostering self-regulation?. J Pers Soc Psychol.

[32] Lepore SJ, Greenberg MA, Bruno M, Smyth JM, Lepore SJ, Smyth JM (2002). Expressive writing and health: self-regulation of emotion-related experience, physiology, and behavior. The writing cure: how expressive writing promotes health and emotional well-being.

[33] Pennebaker JW, Boyd RL, Jordan K, Blackburn K (2015). The development and psychometric properties of LIWC2015.

[34] Chung C, Pennebaker JW. The psychological functions of function words. Social Communication. 2007:343–59.

[35] Pennebaker JW, Mayne TJ, Francis ME (1997). Linguistic predictors of adaptive bereavement. J Pers Soc Psychol.

[36] Pennebaker JW, Mehl MR, Niederhoffer KG (2003). Psychological aspects of natural language use: our words. Our Selves Annual Review of Psychology.

[37] Pennebaker JW, Francis ME, Booth RJ (2001). Linguistic inquiry and word count: LIWC 2001.

[38] Francis ME, Pennebaker JW (1993). Linguistic inquiry and word count. Technical Report.

[39] Moher D, Liberati A, Tetzlaff J, Altman DG, Group P (2009). Preferred reporting items for systematic reviews and meta-analyses: the PRISMA statement. PLoS Med.

[40] Niles AN, Haltom KEB, Mulvenna CM, Lieberman MD, Stanton AL (2014). Randomized controlled trial of expressive writing for psychological and physical health: the moderating role of emotional expressivity. Anxiety Stress Coping.

[41] Higgins J. P. T., Altman D. G., Gotzsche P. C., Juni P., Moher D., Oxman A. D., Savovic J., Schulz K. F., Weeks L., Sterne J. A. C. (2011). The Cochrane Collaboration's tool for assessing risk of bias in randomised trials. BMJ.

[42] Pluye P, Robert E, Cargo M, Bartlett G, O’cathain A, Griffiths F (2011). Proposal: a mixed methods appraisal tool for systematic mixed studies reviews.

[43] Schünemann HJ, Oxman AD, Higgins JPT, Vist GE, Glasziou P, Akl E, JPT H, Churchill R, Chandler J, Cumpston MS (2017). Chapter 11: Completing ‘Summary of findings’ tables and grading the confidence in or quality of the evidence. Cochrane Handbook for Systematic Reviews of Interventions version 520: Cochrane.

[44] Kavanagh BP (2009). The GRADE system for rating clinical guidelines. PLoS Med.

[45] Dijkers M (2013). Introducing GRADE: a systematic approach to rating evidence in systematic reviews and to guideline development. KT Update.

[46] Review Manager (RevMan). [Computer program] (2014). Version 5.3. Copenhagen: The Nordic Cochrane Centre. The Nordic Cochrane Centre TCC.

[47] Deeks JJ, Higgins JP, Altman DG. Analysing data and undertaking meta‐analyses. Cochrane handbook for systematic reviews of interventions version 5.1.0 (updated March 2011). The Cochrane Collaboration; 2011.

[48] Bruera E, Willey J, Cohen M, Palmer JL (2008). Expressive writing in patients receiving palliative care: a feasibility study. J Palliat Med.

[49] Low CA, Stanton AL, Bower JE, Gyllenhammer L (2010). A randomized controlled trial of emotionally expressive writing for women with metastatic breast cancer. Health Psychol.

[50] Mosher CE, DuHamel KN, Lam J, Dickler M, Li Y, Massie MJ (2012). Randomised trial of expressive writing for distressed metastatic breast cancer patients. Psychol Health.

[51] Imrie S, Troop NA (2012). A pilot study on the effects and feasibility of compassion-focused expressive writing in day hospice patients. Palliative and Supportive Care.

[52] Laccetti M (2007). Expressive writing in women with advanced breast Cancer. Oncol Nurs Forum.

[53] Radloff LS (1977). The CES-D scale: a self-report depression scale for research in the general population. Appl Psychol Meas.

[54] Roth AJ, Kornblith AB, Batel-Copel L, Peabody E, Scher HI, Holland JC (1998). Rapid screening for psychologic distress in men with prostate carcinoma. Cancer..

[55] Yellen SB, Cella DF, Webster K, Blendowski C, Kaplan E (1997). Measuring fatigue and other anemia-related symptoms with the functional assessment of Cancer therapy (FACT) measurement system. J Pain Symptom Manag.

[56] Zigmond A. S., Snaith R. P. (1983). The Hospital Anxiety and Depression Scale. Acta Psychiatrica Scandinavica.

[57] Horowitz M, Wilner N, Alvarez W (1979). Impact of event scale: a measure of subjective stress. Psychosom Med.

[58] McNair D, Lorr M, Droppleman L (1981). Profile of mood states: EdITS manual.

[59] Buysse DJ, Reynolds CF, Monk TH, Berman SR, Kupfer DJ (1989). The Pittsburgh sleep quality index: a new instrument for psychiatric practice and research. Psychiatry Res.

[60] Spielberger C (1983). Assessment of anger: the state-trait anger expression scale. Advances in Personality Assessment.

[61] Brady MJ, Cella DF, Mo F, Bonomi AE, Tulsky DS, Lloyd SR (1997). Reliability and validity of the functional assessment of Cancer therapy-breast quality-of-life instrument. J Clin Oncol.

[62] Smyth JM (1998). Written emotional expression: effect sizes, outcome types, and moderating variables. J Consult Clin Psychol.

[63] Forstmeier W, Wagenmakers E-J, Parker TH (2016). Detecting and avoiding likely false-positive findings - a practical guide. Biol Rev.

[64] Tausczik YR, Pennebaker JW (2010). The psychological meaning of words: LIWC and computerized text analysis methods. J Lang Soc Psychol.

[65] Reinhold M, Bürkner PC, Holling H (2018). Effects of expressive writing on depressive symptoms—a meta-analysis. Clin Psychol Sci Pract.

[66] Willmott L, Harris P, Gellaitry G, Cooper V, Horne R (2011). The effects of expressive writing following first myocardial infarction: a randomized controlled trial. Health Psychol.

[67] Zakowski SG, Ramati A, Morton C, Johnson P, Flanigan R (2004). Written emotional disclosure buffers the effects of social constraints on distress among cancer patients. Health Psychol.

[68] Gellaitry G, Peters K, Bloomfield D, Horne R (2010). Narrowing the gap: the effects of an expressive writing intervention on perceptions ofactualandidealemotional support in women who have completed treatment for early stage breast cancer. Psycho-Oncology..

[69] Flemming K (2007). The knowledge base for evidence-based nursing: a role for mixed methods research?. Adv Nurs Sci.

