# Supplementary material for: Expressive writing as a therapeutic intervention for people with advanced disease: a systematic review
Source: BMC Palliat Care. 2019 Aug 2;18:65. doi: 10.1186/s12904-019-0449-y (PMC6676535; doi:10.1186/s12904-019-0449-y)
Supplement: Supplementary file 2 — Table S2: Risk of bias assessment across RCTS. (DOCX 32 kb) [file 12904_2019_449_MOESM2_ESM.docx]

# Additional file 2: Risk of bias assessment across RCTS

| **Study** | **Random sequence generation** | **Allocation concealment** | **Blinding of participants and personnel** | **Blinding of outcome assessment** | **Incomplete outcome data** | **Sample size** |
| --- | --- | --- | --- | --- | --- | --- |
| Bruera (2008) | ? | ? | ? | ? | – | – |
| de Moor (2002) | + | ? | ? | ? | + | – |
| Low (2010) | + | + | ? | ? | + | – |
| Mosher (2012) | + | ? | ? | ? | + | – |

***Note.*** + = low risk of bias; – = high risk of bias; ? = unclear risk of bias
