# Supplementary material for: Expressive writing as a therapeutic intervention for people with advanced disease: a systematic review
Source: BMC Palliat Care. 2019 Aug 2;18:65. doi: 10.1186/s12904-019-0449-y (PMC6676535; doi:10.1186/s12904-019-0449-y)
Supplement: Supplementary file 3 — Table s3: Results of MMAT quality appraisal. (DOCX 14 kb) [file 12904_2019_449_MOESM3_ESM.docx]

**Additional file 3: Results of MMAT quality appraisal**

| **Imrie and Troop 2012** | | | | |
| --- | --- | --- | --- | --- |
|  | **Methodological quality criteria** | **Responses** | | |
|  |  | **Yes** | **No** | **Unclear** |
| Screening questions | Are there clear qualitative and quantitative research questions (or objectives*), or a clear mixed methods question (or objective*)? |  |  |  |
|  | Do the collected data allow address the research question (objective)? E.g., consider whether the follow-up period is long enough for the outcome to occur (for longitudinal studies or study components). |  |  |  |
| Quantitative non- randomised criteria | Are participants (organisations) recruited in a way that minimises selection bias? |  |  |  |
|  | Are measurements appropriate (clear origin, or validity known, or standard instrument; and absence of contamination between groups when appropriate) regarding the exposure/intervention and outcomes? |  |  |  |
|  | In the groups being compared (exposed vs. non-exposed; with intervention vs. without; cases vs. controls), are the participants comparable, or do researchers take into account (control for) the difference between these groups? |  |  |  |
|  | Are there complete outcome data (80% or above), and, when applicable, an acceptable response rate (60% or above), or an acceptable follow-up rate for cohort studies (depending on the duration of follow-up)? |  |  |  |
| **Total score** |  | **75%***** | | |
|  |  |  | | |
|  |  |  | | |
| **Laccetti 2007** | | | | |
| Screening questions | Are there clear qualitative and quantitative research questions (or objectives*), or a clear mixed methods question (or objective*)? |  |  |  |
|  | Do the collected data allow address the research question (objective)? E.g., consider whether the follow-up period is long enough for the outcome to occur (for longitudinal studies or study components). |  |  |  |
| Quantitative descriptive criteria | Is the sampling strategy relevant to address the quantitative research question (quantitative aspect of the mixed methods question)? |  |  |  |
|  | Is the sample representative of the population under study? |  |  |  |
|  | Are measurements appropriate (clear origin, or validity known, or standard instrument)? |  |  |  |
|  | Is there an acceptable response rate (60% or above)? |  |  |  |
| **Total score** |  | **50%**** | | |

***Note. 75%****** = Descriptors evaluating the overall quality score. *=25%, **=50%, ***=75%, ****=100%; Objective*=Mixed study designs have either research questions or research objectives
