# Supplementary material for: Expressive writing as a therapeutic intervention for people with advanced disease: a systematic review
Source: BMC Palliat Care. 2019 Aug 2;18:65. doi: 10.1186/s12904-019-0449-y (PMC6676535; doi:10.1186/s12904-019-0449-y)
Supplement: Supplementary file 4 — Results of the meta-analysis for the outcomes of interest. Figure S1. Pooled data comparing the effectiveness of EW on sleep (sleep quality/sleep duration) compared with the control task. Figure S2. Pooled data comparing the effectiveness of EW on depression compared with the control task. Figure S3. Pooled data comparing the effectiveness of EW on distress compared with the control task. (DOCX 31 kb) [file 12904_2019_449_MOESM4_ESM.docx]

# Additional file 4: Results of the meta-analysis for the outcomes of interest


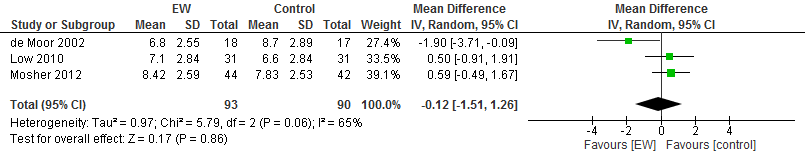


**Figure S1.** Pooled data comparing the effectiveness of EW on sleep (sleep quality/sleep duration) compared with the control task


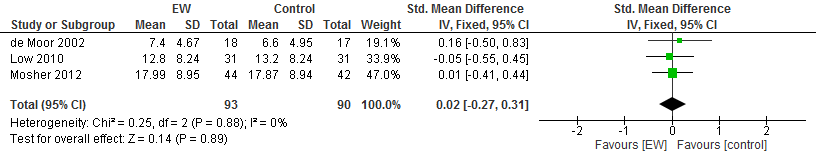


**Figure S2.** Pooled data comparing the effectiveness of EW on depression compared with the control task


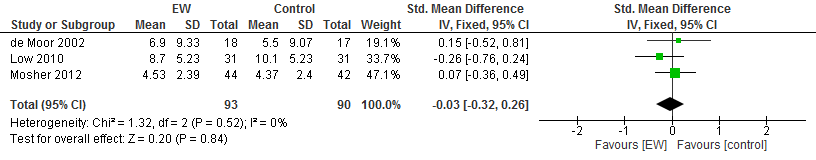


**Figure S3.** Pooled data comparing the effectiveness of EW on distress compared with the control task
